# Crystal structure of *N*-(2-{[2,6-bis­(2,2,2-tri­fluoro­acetamido)­phen­yl]disulfan­yl}-3-(2,2,2-tri­fluoro­acetamido)­phen­yl)-2,2,2-tri­fluoro­acetamide

**DOI:** 10.1107/S2056989015014231

**Published:** 2015-08-06

**Authors:** Dennis Awasabisah, Douglas R. Powell, George B. Richter-Addo

**Affiliations:** a Department of Chemistry and Biochemistry, University of Oklahoma, 101 Stephenson Pkwy, Norman, OK 73019, USA

**Keywords:** crystal structure, diaryl di­sulfide, S—S bonds, N—H⋯S inter­actions, N—H⋯F inter­actions, C—H⋯O inter­actions.

## Abstract

The title compound, C_20_H_10_F_12_N_4_O_4_S_2_, is an organic diaryl di­sulfide compound with tri­fluoro­acetamide substituents at the *ortho*-positions of each benzene ring. There are two mol­ecules (labeled *A* and *B*) in the asymmetric unit. The F atoms of three of the –CF_3_ groups exhibit rotational disorder over two positions each. The S—S bond distances are 2.0914 (7) and 2.0827 (6) Å for mol­ecules *A* and *B*, respectively. The dihedral angle between the S—S—C and S—C—C planes is 103.05 (15)° for mol­ecule *A* and 104.09 (15)° for mol­ecule *B*. The three-dimensional supra­molecular architecture of the crystal is sustained by numerous N—H⋯O, N—H⋯S and C—H⋯O inter­actions.

## Related literature   

For the synthesis of di­thio­bis­(*N*-phenyl­amide) compounds, see: Ueyama *et al.* (1995 ▸); Lumb *et al.* (2014 ▸). For related crystal structures, see: Ueyama *et al.* (1995 ▸); Raftery *et al.* (2009 ▸). For applications of the title compound and related compounds, see: Klingele *et al.* (2013 ▸); Xu *et al.* (2006 ▸); Enemark & Cooney (2004 ▸); Yu *et al.* (2008 ▸); Smith *et al.* (2005 ▸); Ueyama *et al.* (1995 ▸, 1998 ▸); Reichardt *et al.* (2003 ▸); Dance (1986 ▸).
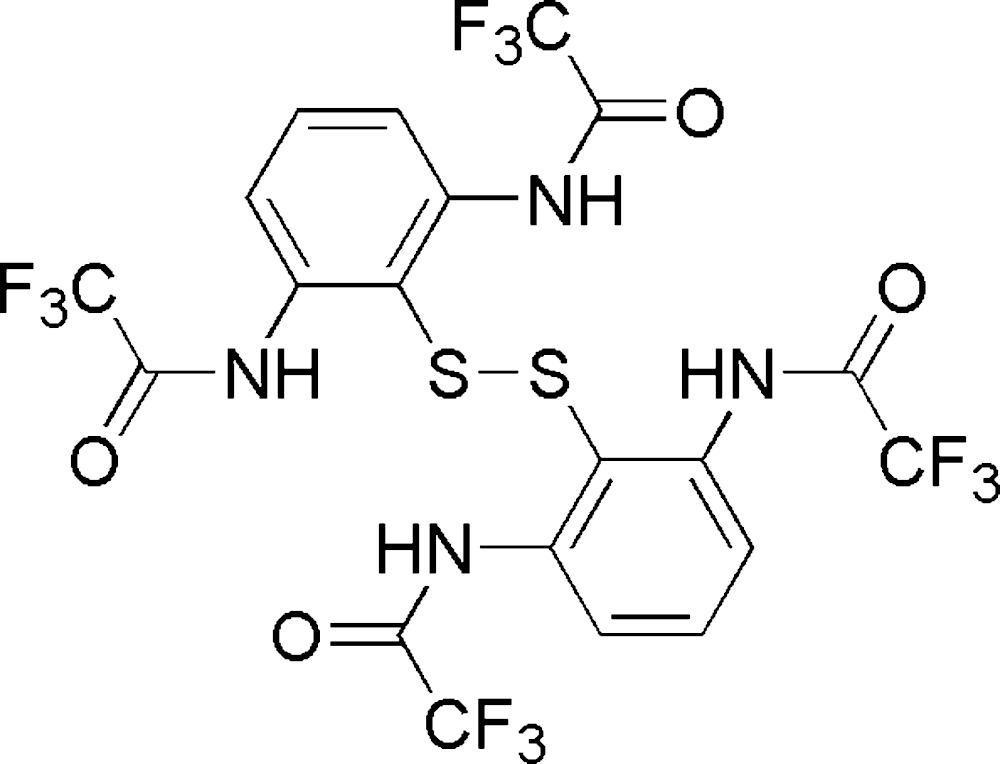


## Experimental   

### Crystal data  

C_20_H_10_F_12_N_4_O_4_S_2_
*M* *_r_* = 662.44
Monoclinic, 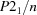

*a* = 19.0538 (10) Å
*b* = 13.1466 (7) Å
*c* = 19.961 (1) Å
β = 96.0042 (9)°
*V* = 4972.7 (4) Å^3^
*Z* = 8
Mo *K*α radiation
μ = 0.34 mm^−1^
*T* = 100 K
0.65 × 0.25 × 0.14 mm

### Data collection  

Bruker APEX CCD diffractometer
Absorption correction: multi-scan (*SADABS*; Bruker, 2002 ▸) *T*_min_ = 0.808, *T*_max_ = 0.954
91119 measured reflections
12375 independent reflections
10553 reflections with *I* > 2σ(*I*)
*R*_int_ = 0.033

### Refinement  

*R*[*F* ^2^ > 2σ(*F* ^2^)] = 0.047
*wR*(*F* ^2^) = 0.135
*S* = 1.00
12375 reflections
865 parameters
393 restraints
H atoms treated by a mixture of independent and constrained refinement
Δρ_max_ = 1.37 e Å^−3^
Δρ_min_ = −1.09 e Å^−3^

### 

Data collection: *SMART* (Bruker, 2007 ▸); cell refinement: *SAINT*; data reduction: *SAINT* (Bruker, 2007 ▸); program(s) used to solve structure: *SHELXS* (Sheldrick, 2008 ▸); program(s) used to refine structure: *SHELXL2014* (Sheldrick, 2015 ▸); molecular graphics: *SHELXL2002*; software used to prepare material for publication: *SHELXL2014*.

## Supplementary Material

Crystal structure: contains datablock(s) I, New_Global_Publ_Block. DOI: 10.1107/S2056989015014231/hb7457sup1.cif

Structure factors: contains datablock(s) I. DOI: 10.1107/S2056989015014231/hb7457Isup2.hkl

Supporting information file. DOI: 10.1107/S2056989015014231/hb7457Isup3.cml

**Fig. 1.**
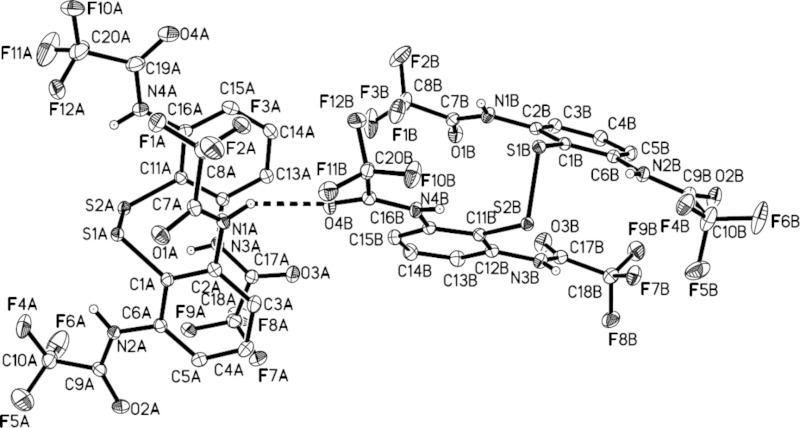
The molecular structure of the title compound, showing displacement ellipsoids drawn at the 50% probability level. Aromatic H atoms and disordered groups have been omitted for clarity.

. DOI: 10.1107/S2056989015014231/hb7457fig1.tif
The mol­ecular structure of the title compound, showing displacement ellipsoids drawn at the 50% probability level. Aromatic H atoms and disordered groups have been omitted for clarity.

**Fig. 2.**
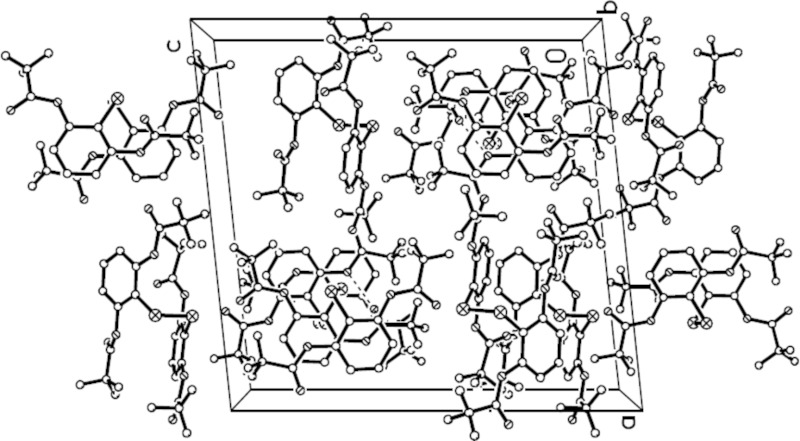
The packing diagram.

. DOI: 10.1107/S2056989015014231/hb7457fig2.tif
The packing diagram.

CCDC reference: 1415414

Additional supporting information:  crystallographic information; 3D view; checkCIF report

## Figures and Tables

**Table 1:** Hydrogen-bond geometry (, )

*D*H*A*	*D*H	H*A*	*D* *A*	*D*H*A*
N1*A*H1*AN*O4*B*	0.89(3)	2.19(3)	2.988(2)	149(2)
N2*A*H2*AN*S1*A*	0.84(3)	2.42(3)	2.9425(18)	121(2)
N2*A*H2*AN*S2*A*	0.84(3)	2.96(3)	3.4543(18)	120(2)
N3*A*H3*AN*O1*A* ^i^	0.84(3)	2.12(3)	2.857(2)	146(2)
C5*A*H5*A*O2*A*	0.95	2.34	2.952(3)	122
C13*A*H13*A*F5*B* ^ii^	0.95	2.54	3.207(4)	127
C15*A*H15*A*O4*A*	0.95	2.25	2.883(3)	123
N1*B*H1*BN*O2*A* ^iii^	0.80(3)	2.42(3)	2.983(2)	128(2)
N3*B*H3*BN*O1*B* ^iv^	0.79(3)	2.24(3)	2.848(2)	135(3)
N4*B*H4*BN*O3*B* ^iv^	0.85(3)	2.47(3)	3.032(2)	125(2)
C5*B*H5*B*O2*B*	0.95	2.27	2.896(3)	122
C15*B*H15*B*O3*A*	0.95	2.56	3.229(3)	128
C15*B*H15*B*O4*B*	0.95	2.27	2.898(3)	123

Symmetry codes: (i) 
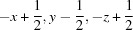
; (ii) 
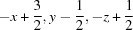
; (iii) 
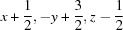
; (iv) 
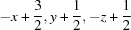
.

## supplementary crystallographic information

### S1. Introduction

Thiol­ate coordination to metal centers are common in metalloproteins (Enemark *et al.*, 2004), and several crystal structures of thiol­ate complexes are known (Klingele *et al.*, 2013; Dance, 1986). Further, di­thio-bis-*N*-phenyl compounds such as the title compound, C_20_H_10_F_12_N_4_O_4_S_2_, have often been used as precursors for the synthesis of metal thiol­ate complexes (Yu *et al.*, 2008; Smith *et al.*, 2005; Ueyama *et al.*, 1998; Ueyama *et al.*, 1996; Xu *et al.*, 2006). Only a handful of crystal structures of di­thio-bis-*N*-phenyl­amide compounds are known (Ueyama *et al.*, 1995; Raftery *et al.*, 2009). We now report the crystal structure of the known compound 2,2'-di­thio-bis­(N-phenyl-2,2,2-tri­fluoro­acetamide) (Ueyama *et al.*, 1998; Ueyama, *et al.*, 1995) (Fig. 1). There are two formula units (molecule A and molecule B) per asymmetric unit of the cell. The S–S bond distances are 2.0914 (7) Å for molecule A, and 2.0827 (6) Å, for molecule B. The dihedral angles between the S–S–C planes and the S–C–C planes are 103.05 (15) ° for molecule A, and 104.09 (15) ° for molecule B.

### S2. Experimental

The title compound, C_20_H_10_F_12_N_4_O_4_S_2_, was prepared as reported in the literature (Ueyama *et al.*, 1998; Ueyama, *et al.*, 1995) to give 90% isolated yield of the yellow product. IR (KBr, cm^-1^): ν_NH_ = 3362, 3335; ν_CO_ = 1741, 1730. ^19^F NMR (CDCl_3_, ppm): δ –75.61 (s, C*F*_3_). ^1^H NMR (CDCl_3_, ppm): δ 8.59 (s, 4H, N*H*); 8.21 (d, *J* = 8.7 Hz, 4H, phenyl-*H*); 7.62 (t, *J* = 8.7 Hz, 2H, phenyl-*H*). Single crystals of the compound were obtained by a slow evaporation of a methyl­ene chloride/ hexane (2:1) solution of the compound.

### S3. Refinement

H atoms were located geometrically and refined using a riding model on their parent atoms, with C–H = 0.95 Å for aromatic, with U_iso_(H) = 1.2–1.5U_eq_(C). The F atoms of three of the CF_3_ groups exhibit rotational disorder over two positions each. The occupancies of atoms F4A - F6A were refined to 0.538 (10) and 0.462 (10) for the unprimed and primed atoms. The occupancies of atoms F10A – F12A were refined to 0.509 (7) and 0.491 (7) for the A and C labeled atoms. The occupancies of atoms F4B – F6B were refined to 0.658 (7) and 0.342 (7) for the unprimed and primed atoms. Restraints on the positional and displacement parameters of the disordered atoms were required. The final difference map had maxima and minima of 1.375 and -1.087 e/Å^3^, respectively, which were located close to the disordered F atoms. Specifically, the largest peak was located close to F12A and the smallest hole was located close to F12C.

### Crystal data

**Table:**

C_20_H_10_F_12_N_4_O_4_S_2_	*F*(000) = 2640
*M**_r_* = 662.44	*D*_x_ = 1.770 Mg m^−^^3^
Monoclinic, *P*2_1_/*n*	Mo *K*α radiation, λ = 0.71073 Å
*a* = 19.0538 (10) Å	Cell parameters from 7979 reflections
*b* = 13.1466 (7) Å	θ = 2.6–28.2°
*c* = 19.961 (1) Å	µ = 0.34 mm^−^^1^
β = 96.0042 (9)°	*T* = 100 K
*V* = 4972.7 (4) Å^3^	Prism, black
*Z* = 8	0.65 × 0.25 × 0.14 mm

### Data collection

**Table:**

Bruker APEX CCD diffractometer	10553 reflections with *I* > 2σ(*I*)
φ and ω scans	*R*_int_ = 0.033
Absorption correction: multi-scan (*SADABS*; Bruker, 2002)	θ_max_ = 28.3°, θ_min_ = 1.4°
*T*_min_ = 0.808, *T*_max_ = 0.954	*h* = −25→25
91119 measured reflections	*k* = −17→17
12375 independent reflections	*l* = −26→26

### Refinement

**Table:**

Refinement on *F*^2^	393 restraints
Least-squares matrix: full	Hydrogen site location: mixed
*R*[*F*^2^ > 2σ(*F*^2^)] = 0.047	H atoms treated by a mixture of independent and constrained refinement
*wR*(*F*^2^) = 0.135	*w* = 1/[σ^2^(*F*_o_^2^) + (0.076*P*)^2^ + 6.*P*] where *P* = (*F*_o_^2^ + 2*F*_c_^2^)/3
*S* = 1.00	(Δ/σ)_max_ = 0.012
12375 reflections	Δρ_max_ = 1.37 e Å^−^^3^
865 parameters	Δρ_min_ = −1.09 e Å^−^^3^

### Special details

**Table:**

Geometry. All e.s.d.'s (except the e.s.d. in the dihedral angle between two l.s. planes) are estimated using the full covariance matrix. The cell e.s.d.'s are taken into account individually in the estimation of e.s.d.'s in distances, angles and torsion angles; correlations between e.s.d.'s in cell parameters are only used when they are defined by crystal symmetry. An approximate (isotropic) treatment of cell e.s.d.'s is used for estimating e.s.d.'s involving l.s. planes.

### Fractional atomic coordinates and isotropic or equivalent isotropic displacement parameters (Å^2^)

**Table:**

	*x*	*y*	*z*	*U*_iso_*/*U*_eq_	Occ. (<1)
S1A	0.19690 (2)	0.84332 (4)	0.20932 (2)	0.02061 (10)	
S2A	0.20039 (2)	0.68776 (4)	0.23136 (2)	0.02119 (10)	
F1A	0.23377 (7)	0.89398 (14)	0.02002 (7)	0.0434 (4)	
F2A	0.30695 (9)	1.01596 (12)	0.00909 (7)	0.0431 (4)	
F3A	0.34531 (7)	0.86410 (11)	0.03116 (7)	0.0357 (3)	
F4A	0.0781 (2)	0.8272 (5)	0.36999 (16)	0.0461 (11)	0.538 (10)
F5A	0.0872 (3)	0.8762 (5)	0.4716 (3)	0.0753 (15)	0.538 (10)
F6A	0.1246 (3)	0.7287 (3)	0.4482 (4)	0.0715 (16)	0.538 (10)
F4A'	0.0882 (3)	0.7822 (7)	0.3719 (2)	0.0701 (17)	0.462 (10)
F5A'	0.1213 (3)	0.7535 (4)	0.4753 (3)	0.0546 (14)	0.462 (10)
F6A'	0.0761 (3)	0.8950 (3)	0.4463 (4)	0.0617 (14)	0.462 (10)
F7A	0.42433 (8)	0.74860 (13)	0.45323 (7)	0.0437 (4)	
F8A	0.39524 (10)	0.59029 (12)	0.45287 (8)	0.0501 (4)	
F9A	0.31648 (7)	0.70381 (10)	0.42694 (6)	0.0302 (3)	
F10A	0.1024 (3)	0.6932 (5)	−0.07119 (14)	0.0464 (13)	0.509 (7)
F11A	0.0700 (2)	0.6129 (5)	0.0155 (3)	0.0885 (17)	0.509 (7)
F12A	0.09429 (19)	0.7685 (3)	0.0215 (2)	0.0633 (13)	0.509 (7)
F10C	0.1026 (3)	0.7185 (5)	−0.06545 (18)	0.0508 (14)	0.491 (7)
F11C	0.0893 (2)	0.5786 (3)	−0.0144 (2)	0.0672 (13)	0.491 (7)
F12C	0.0773 (2)	0.7136 (6)	0.0395 (2)	0.0849 (16)	0.491 (7)
O1A	0.25566 (9)	1.02169 (12)	0.13452 (8)	0.0315 (3)	
O2A	0.22941 (8)	0.89280 (12)	0.47115 (7)	0.0281 (3)	
O3A	0.45890 (9)	0.68634 (17)	0.33375 (9)	0.0423 (4)	
O4A	0.23430 (10)	0.64932 (16)	−0.02549 (8)	0.0431 (4)	
N1A	0.33815 (9)	0.89887 (13)	0.16183 (8)	0.0227 (3)	
H1AN	0.3731 (14)	0.862 (2)	0.1483 (13)	0.027*	
N2A	0.20760 (9)	0.87028 (14)	0.35637 (9)	0.0234 (3)	
H2AN	0.1753 (15)	0.854 (2)	0.3262 (14)	0.028*	
N3A	0.34280 (9)	0.64515 (13)	0.30362 (8)	0.0226 (3)	
H3AN	0.3053 (15)	0.633 (2)	0.3209 (13)	0.027*	
N4A	0.20769 (10)	0.66134 (15)	0.08449 (9)	0.0276 (4)	
H4AN	0.1734 (16)	0.673 (2)	0.1056 (15)	0.033*	
C1A	0.27294 (10)	0.88575 (14)	0.26082 (9)	0.0199 (3)	
C2A	0.33560 (10)	0.90799 (15)	0.23293 (10)	0.0216 (4)	
C3A	0.39579 (11)	0.93739 (16)	0.27373 (11)	0.0269 (4)	
H3A	0.4382	0.9522	0.2545	0.032*	
C4A	0.39344 (11)	0.94497 (17)	0.34279 (11)	0.0281 (4)	
H4A	0.4348	0.9640	0.3706	0.034*	
C5A	0.33191 (11)	0.92536 (15)	0.37207 (10)	0.0247 (4)	
H5A	0.3309	0.9318	0.4194	0.030*	
C6A	0.27135 (10)	0.89603 (14)	0.33090 (10)	0.0207 (4)	
C7A	0.29602 (10)	0.95652 (16)	0.11896 (10)	0.0241 (4)	
C8A	0.29648 (11)	0.93193 (18)	0.04348 (11)	0.0285 (4)	
C9A	0.19261 (11)	0.86697 (15)	0.42072 (10)	0.0244 (4)	
C10A	0.11892 (12)	0.82341 (16)	0.42756 (9)	0.0365 (5)	
C11A	0.27654 (10)	0.65281 (14)	0.19298 (10)	0.0206 (4)	
C12A	0.34090 (10)	0.63600 (14)	0.23269 (10)	0.0207 (4)	
C13A	0.40107 (11)	0.61034 (16)	0.20217 (10)	0.0248 (4)	
H13A	0.4444	0.5968	0.2287	0.030*	
C14A	0.39672 (12)	0.60491 (16)	0.13246 (11)	0.0279 (4)	
H14A	0.4381	0.5891	0.1118	0.033*	
C15A	0.33420 (12)	0.62171 (16)	0.09191 (11)	0.0277 (4)	
H15A	0.3328	0.6177	0.0443	0.033*	
C16A	0.27341 (11)	0.64465 (15)	0.12229 (10)	0.0232 (4)	
C17A	0.39951 (11)	0.67082 (16)	0.34650 (10)	0.0250 (4)	
C18A	0.38331 (12)	0.67836 (17)	0.42094 (11)	0.0288 (4)	
C19A	0.19369 (13)	0.66172 (19)	0.01649 (11)	0.0354 (5)	
C20A	0.11558 (16)	0.6769 (2)	−0.00550 (10)	0.0597 (8)	
S1B	0.76647 (2)	0.76045 (4)	0.08659 (2)	0.01966 (10)	
S2B	0.76118 (2)	0.85065 (3)	0.17168 (2)	0.01906 (10)	
F1B	0.57541 (7)	0.59534 (12)	0.09799 (9)	0.0438 (4)	
F2B	0.57993 (9)	0.44061 (15)	0.06322 (10)	0.0607 (5)	
F3B	0.55214 (8)	0.47312 (16)	0.16212 (10)	0.0610 (5)	
F4B	0.94973 (17)	0.9556 (3)	0.0933 (2)	0.0350 (8)	0.658 (7)
F5B	0.9957 (2)	0.9478 (2)	0.19691 (12)	0.0541 (9)	0.658 (7)
F6B	1.06209 (11)	0.9342 (2)	0.1184 (2)	0.0579 (10)	0.658 (7)
F4B'	0.9405 (3)	0.9559 (5)	0.1081 (4)	0.0391 (18)	0.342 (7)
F5B'	1.0320 (4)	0.9589 (4)	0.1824 (3)	0.0731 (18)	0.342 (7)
F6B'	1.0413 (4)	0.9165 (4)	0.0809 (4)	0.0782 (18)	0.342 (7)
F7B	0.95251 (7)	0.75811 (11)	0.28940 (7)	0.0338 (3)	
F8B	0.94940 (8)	0.70254 (12)	0.39064 (7)	0.0394 (3)	
F9B	0.98062 (7)	0.60249 (12)	0.31369 (8)	0.0405 (3)	
F10B	0.57446 (7)	0.96551 (14)	0.05725 (8)	0.0468 (4)	
F11B	0.46691 (7)	0.97928 (10)	0.07994 (7)	0.0337 (3)	
F12B	0.49804 (7)	0.85087 (12)	0.02331 (7)	0.0392 (3)	
O1B	0.68797 (8)	0.43192 (12)	0.18963 (8)	0.0310 (3)	
O2B	1.03946 (8)	0.74409 (13)	0.16381 (10)	0.0368 (4)	
O3B	0.84306 (9)	0.55663 (12)	0.32275 (10)	0.0395 (4)	
O4B	0.48416 (8)	0.81527 (13)	0.16359 (8)	0.0315 (3)	
N1B	0.71462 (9)	0.55321 (14)	0.11416 (9)	0.0241 (3)	
H1BN	0.6955 (14)	0.587 (2)	0.0841 (14)	0.029*	
N2B	0.92272 (9)	0.76337 (13)	0.12265 (9)	0.0233 (3)	
H2BN	0.8947 (15)	0.808 (2)	0.1056 (13)	0.028*	
N3B	0.81672 (9)	0.71875 (14)	0.28596 (9)	0.0236 (3)	
H3BN	0.8331 (15)	0.772 (2)	0.2790 (13)	0.028*	
N4B	0.60340 (9)	0.84012 (13)	0.15931 (9)	0.0225 (3)	
H4BN	0.6307 (14)	0.880 (2)	0.1408 (13)	0.027*	
C1B	0.81979 (10)	0.65657 (14)	0.11839 (9)	0.0196 (3)	
C2B	0.78839 (10)	0.56231 (15)	0.12953 (9)	0.0205 (4)	
C3B	0.82932 (11)	0.47958 (15)	0.15394 (10)	0.0244 (4)	
H3B	0.8080	0.4158	0.1612	0.029*	
C4B	0.90147 (11)	0.49212 (16)	0.16734 (11)	0.0278 (4)	
H4B	0.9295	0.4360	0.1840	0.033*	
C5B	0.93418 (11)	0.58450 (16)	0.15708 (11)	0.0263 (4)	
H5B	0.9838	0.5914	0.1669	0.032*	
C6B	0.89356 (10)	0.66657 (15)	0.13236 (10)	0.0216 (4)	
C7B	0.67175 (11)	0.49148 (15)	0.14464 (10)	0.0230 (4)	
C8B	0.59362 (12)	0.49961 (18)	0.11635 (13)	0.0336 (5)	
C9B	0.98979 (10)	0.79415 (16)	0.14079 (10)	0.0258 (4)	
C10B	0.99935 (9)	0.90797 (19)	0.13293 (11)	0.0434 (6)	
C11B	0.70913 (10)	0.77559 (14)	0.22181 (10)	0.0202 (3)	
C12B	0.74232 (10)	0.71432 (15)	0.27340 (10)	0.0220 (4)	
C13B	0.70246 (12)	0.65474 (16)	0.31323 (11)	0.0279 (4)	
H13B	0.7249	0.6118	0.3473	0.033*	
C14B	0.62966 (12)	0.65928 (17)	0.30223 (11)	0.0293 (4)	
H14B	0.6024	0.6191	0.3295	0.035*	
C15B	0.59506 (11)	0.72048 (16)	0.25274 (10)	0.0254 (4)	
H15B	0.5450	0.7229	0.2466	0.030*	
C16B	0.63503 (10)	0.77851 (14)	0.21210 (10)	0.0209 (4)	
C17B	0.85908 (11)	0.64340 (16)	0.31062 (10)	0.0255 (4)	
C18B	0.93663 (12)	0.67737 (17)	0.32606 (11)	0.0287 (4)	
C19B	0.53419 (10)	0.85090 (15)	0.13889 (10)	0.0225 (4)	
C20B	0.51895 (11)	0.91349 (18)	0.07366 (10)	0.0273 (4)	

### Atomic displacement parameters (Å^2^)

**Table:**

	*U*^11^	*U*^22^	*U*^33^	*U*^12^	*U*^13^	*U*^23^
S1A	0.0177 (2)	0.0242 (2)	0.0198 (2)	0.00157 (16)	0.00127 (16)	−0.00039 (16)
S2A	0.0180 (2)	0.0237 (2)	0.0220 (2)	−0.00232 (17)	0.00287 (16)	−0.00006 (17)
F1A	0.0276 (7)	0.0669 (10)	0.0350 (7)	−0.0050 (7)	−0.0001 (6)	−0.0083 (7)
F2A	0.0544 (9)	0.0457 (8)	0.0314 (7)	0.0047 (7)	0.0155 (6)	0.0133 (6)
F3A	0.0334 (7)	0.0455 (8)	0.0289 (7)	0.0092 (6)	0.0060 (5)	−0.0061 (6)
F4A	0.0219 (14)	0.077 (3)	0.0389 (16)	−0.0043 (16)	0.0008 (11)	0.0146 (15)
F5A	0.059 (2)	0.121 (3)	0.053 (3)	−0.016 (2)	0.038 (2)	−0.023 (2)
F6A	0.055 (2)	0.068 (2)	0.088 (3)	−0.0292 (19)	−0.010 (2)	0.033 (2)
F4A'	0.039 (2)	0.130 (4)	0.042 (2)	−0.039 (3)	0.0065 (17)	−0.018 (2)
F5A'	0.0361 (19)	0.066 (3)	0.059 (3)	−0.0242 (19)	−0.0043 (19)	0.023 (2)
F6A'	0.040 (2)	0.082 (3)	0.068 (3)	0.0201 (19)	0.034 (2)	0.032 (2)
F7A	0.0360 (8)	0.0631 (10)	0.0304 (7)	−0.0060 (7)	−0.0045 (6)	−0.0148 (7)
F8A	0.0771 (12)	0.0432 (8)	0.0305 (7)	0.0261 (8)	0.0079 (7)	0.0101 (6)
F9A	0.0316 (7)	0.0339 (7)	0.0260 (6)	0.0009 (5)	0.0081 (5)	−0.0031 (5)
F10A	0.062 (2)	0.047 (3)	0.0267 (17)	0.0325 (18)	−0.0101 (15)	−0.0094 (14)
F11A	0.046 (2)	0.140 (4)	0.075 (3)	−0.026 (2)	−0.013 (2)	0.050 (3)
F12A	0.0270 (17)	0.126 (3)	0.038 (2)	0.0045 (19)	0.0092 (14)	−0.022 (2)
F10C	0.063 (3)	0.054 (3)	0.0327 (19)	0.024 (2)	−0.0083 (17)	−0.0093 (17)
F11C	0.035 (2)	0.129 (3)	0.036 (2)	−0.027 (2)	−0.0018 (15)	0.019 (2)
F12C	0.038 (2)	0.180 (4)	0.036 (2)	0.019 (3)	0.0059 (15)	−0.018 (3)
O1A	0.0343 (8)	0.0299 (8)	0.0311 (8)	0.0105 (6)	0.0074 (6)	0.0030 (6)
O2A	0.0347 (8)	0.0295 (7)	0.0200 (7)	−0.0013 (6)	0.0024 (6)	−0.0015 (6)
O3A	0.0231 (8)	0.0704 (13)	0.0340 (9)	−0.0090 (8)	0.0051 (7)	−0.0121 (8)
O4A	0.0459 (10)	0.0608 (12)	0.0224 (8)	−0.0016 (9)	0.0026 (7)	−0.0057 (8)
N1A	0.0203 (8)	0.0262 (8)	0.0224 (8)	0.0025 (6)	0.0057 (6)	0.0005 (6)
N2A	0.0193 (8)	0.0322 (9)	0.0189 (8)	−0.0009 (7)	0.0025 (6)	−0.0024 (6)
N3A	0.0197 (8)	0.0282 (8)	0.0202 (8)	−0.0007 (6)	0.0039 (6)	0.0012 (6)
N4A	0.0281 (9)	0.0331 (9)	0.0211 (8)	−0.0005 (7)	0.0009 (7)	−0.0019 (7)
C1A	0.0186 (8)	0.0195 (8)	0.0216 (9)	0.0013 (7)	0.0011 (7)	−0.0006 (7)
C2A	0.0217 (9)	0.0206 (8)	0.0229 (9)	0.0012 (7)	0.0047 (7)	0.0014 (7)
C3A	0.0206 (9)	0.0299 (10)	0.0303 (10)	−0.0044 (8)	0.0032 (8)	0.0019 (8)
C4A	0.0244 (10)	0.0294 (10)	0.0295 (10)	−0.0065 (8)	−0.0026 (8)	0.0001 (8)
C5A	0.0270 (10)	0.0229 (9)	0.0239 (9)	−0.0024 (7)	0.0009 (7)	−0.0012 (7)
C6A	0.0200 (8)	0.0195 (8)	0.0229 (9)	0.0013 (7)	0.0032 (7)	0.0002 (7)
C7A	0.0218 (9)	0.0258 (9)	0.0254 (9)	0.0000 (7)	0.0061 (7)	0.0022 (7)
C8A	0.0238 (10)	0.0366 (11)	0.0256 (10)	0.0020 (8)	0.0053 (8)	0.0024 (8)
C9A	0.0251 (9)	0.0268 (9)	0.0219 (9)	0.0027 (8)	0.0050 (7)	−0.0005 (7)
C10A	0.0268 (11)	0.0573 (14)	0.0261 (10)	−0.0036 (10)	0.0061 (8)	0.0018 (10)
C11A	0.0204 (9)	0.0192 (8)	0.0226 (9)	−0.0012 (7)	0.0047 (7)	−0.0001 (7)
C12A	0.0216 (9)	0.0194 (8)	0.0216 (9)	−0.0018 (7)	0.0045 (7)	0.0002 (7)
C13A	0.0225 (9)	0.0254 (9)	0.0269 (10)	0.0018 (7)	0.0048 (7)	−0.0004 (8)
C14A	0.0277 (10)	0.0277 (10)	0.0299 (10)	0.0014 (8)	0.0106 (8)	−0.0025 (8)
C15A	0.0342 (11)	0.0273 (10)	0.0226 (9)	0.0002 (8)	0.0074 (8)	−0.0028 (8)
C16A	0.0268 (10)	0.0200 (9)	0.0225 (9)	−0.0023 (7)	0.0009 (7)	−0.0020 (7)
C17A	0.0225 (9)	0.0294 (10)	0.0230 (9)	0.0024 (8)	0.0015 (7)	−0.0011 (8)
C18A	0.0306 (11)	0.0309 (10)	0.0244 (10)	0.0058 (8)	−0.0001 (8)	−0.0005 (8)
C19A	0.0388 (13)	0.0433 (13)	0.0231 (10)	−0.0036 (10)	−0.0019 (9)	−0.0022 (9)
C20A	0.0391 (14)	0.112 (3)	0.0264 (12)	−0.0073 (16)	−0.0034 (10)	−0.0018 (14)
S1B	0.0197 (2)	0.0210 (2)	0.0182 (2)	0.00084 (16)	0.00150 (16)	0.00107 (16)
S2B	0.0181 (2)	0.0179 (2)	0.0214 (2)	−0.00131 (16)	0.00369 (16)	−0.00046 (16)
F1B	0.0259 (7)	0.0403 (8)	0.0630 (10)	0.0011 (6)	−0.0056 (6)	0.0145 (7)
F2B	0.0470 (10)	0.0589 (11)	0.0699 (12)	−0.0105 (8)	−0.0240 (9)	−0.0159 (9)
F3B	0.0246 (7)	0.0814 (13)	0.0776 (13)	−0.0024 (8)	0.0084 (7)	0.0411 (10)
F4B	0.0316 (13)	0.0272 (13)	0.0443 (17)	−0.0061 (10)	−0.0050 (13)	0.0079 (11)
F5B	0.070 (2)	0.0371 (13)	0.0536 (15)	−0.0191 (14)	−0.0006 (13)	−0.0121 (11)
F6B	0.0247 (12)	0.0452 (15)	0.101 (3)	−0.0127 (10)	−0.0059 (13)	0.0268 (16)
F4B'	0.037 (3)	0.031 (3)	0.050 (3)	−0.001 (2)	0.008 (2)	−0.002 (2)
F5B'	0.053 (3)	0.038 (2)	0.118 (4)	−0.013 (2)	−0.037 (3)	−0.012 (3)
F6B'	0.047 (3)	0.061 (3)	0.130 (4)	−0.016 (2)	0.022 (3)	0.027 (3)
F7B	0.0260 (6)	0.0369 (7)	0.0373 (7)	−0.0034 (5)	−0.0021 (5)	0.0104 (6)
F8B	0.0378 (8)	0.0522 (9)	0.0262 (7)	−0.0071 (6)	−0.0062 (6)	0.0001 (6)
F9B	0.0301 (7)	0.0411 (8)	0.0490 (8)	0.0125 (6)	−0.0025 (6)	0.0035 (6)
F10B	0.0280 (7)	0.0736 (11)	0.0374 (8)	−0.0135 (7)	−0.0031 (6)	0.0275 (7)
F11B	0.0342 (7)	0.0328 (7)	0.0328 (7)	0.0054 (5)	−0.0027 (5)	0.0034 (5)
F12B	0.0325 (7)	0.0570 (9)	0.0267 (7)	0.0070 (6)	−0.0026 (5)	−0.0121 (6)
O1B	0.0273 (7)	0.0326 (8)	0.0331 (8)	−0.0003 (6)	0.0036 (6)	0.0113 (6)
O2B	0.0211 (7)	0.0346 (8)	0.0536 (11)	−0.0019 (6)	−0.0017 (7)	0.0048 (7)
O3B	0.0365 (9)	0.0232 (7)	0.0579 (11)	0.0013 (7)	0.0001 (8)	0.0053 (7)
O4B	0.0203 (7)	0.0356 (8)	0.0397 (9)	0.0018 (6)	0.0084 (6)	0.0103 (7)
N1B	0.0204 (8)	0.0258 (8)	0.0251 (8)	−0.0017 (6)	−0.0029 (6)	0.0059 (7)
N2B	0.0191 (8)	0.0209 (8)	0.0298 (9)	−0.0005 (6)	0.0020 (6)	−0.0013 (6)
N3B	0.0229 (8)	0.0220 (8)	0.0254 (8)	−0.0011 (7)	0.0007 (6)	0.0030 (6)
N4B	0.0185 (7)	0.0263 (8)	0.0230 (8)	−0.0038 (6)	0.0032 (6)	0.0035 (6)
C1B	0.0189 (8)	0.0208 (8)	0.0189 (8)	0.0023 (7)	0.0013 (7)	−0.0005 (6)
C2B	0.0201 (9)	0.0227 (9)	0.0187 (8)	0.0000 (7)	0.0013 (7)	−0.0005 (7)
C3B	0.0243 (9)	0.0207 (9)	0.0278 (10)	0.0012 (7)	0.0009 (8)	0.0008 (7)
C4B	0.0251 (10)	0.0232 (9)	0.0346 (11)	0.0049 (8)	0.0009 (8)	0.0005 (8)
C5B	0.0200 (9)	0.0259 (10)	0.0328 (10)	0.0029 (7)	0.0013 (8)	−0.0038 (8)
C6B	0.0208 (9)	0.0221 (9)	0.0223 (9)	−0.0004 (7)	0.0032 (7)	−0.0027 (7)
C7B	0.0233 (9)	0.0213 (9)	0.0242 (9)	−0.0017 (7)	0.0013 (7)	−0.0011 (7)
C8B	0.0247 (10)	0.0338 (11)	0.0412 (12)	−0.0069 (9)	−0.0017 (9)	0.0102 (9)
C9B	0.0209 (9)	0.0278 (10)	0.0287 (10)	−0.0023 (8)	0.0030 (7)	−0.0029 (8)
C10B	0.0256 (11)	0.0307 (11)	0.0716 (17)	−0.0067 (9)	−0.0053 (11)	0.0020 (11)
C11B	0.0208 (9)	0.0187 (8)	0.0216 (9)	−0.0025 (7)	0.0043 (7)	−0.0017 (7)
C12B	0.0224 (9)	0.0213 (9)	0.0223 (9)	−0.0009 (7)	0.0024 (7)	−0.0018 (7)
C13B	0.0310 (11)	0.0278 (10)	0.0251 (10)	−0.0004 (8)	0.0039 (8)	0.0059 (8)
C14B	0.0297 (11)	0.0310 (10)	0.0285 (10)	−0.0056 (8)	0.0091 (8)	0.0057 (8)
C15B	0.0228 (9)	0.0277 (10)	0.0264 (10)	−0.0037 (8)	0.0058 (7)	−0.0006 (8)
C16B	0.0214 (9)	0.0207 (8)	0.0208 (9)	−0.0012 (7)	0.0029 (7)	−0.0018 (7)
C17B	0.0269 (10)	0.0242 (9)	0.0249 (9)	0.0012 (8)	0.0000 (8)	−0.0016 (7)
C18B	0.0283 (10)	0.0293 (10)	0.0273 (10)	0.0023 (8)	−0.0033 (8)	0.0039 (8)
C19B	0.0209 (9)	0.0223 (9)	0.0244 (9)	0.0003 (7)	0.0024 (7)	−0.0023 (7)
C20B	0.0197 (9)	0.0377 (11)	0.0244 (10)	−0.0023 (8)	0.0011 (7)	0.0009 (8)

### Geometric parameters (Å, º)

**Table:**

S1A—C1A	1.7763 (19)	C19A—C20A	1.520 (4)
S1A—S2A	2.0914 (7)	S1B—C1B	1.7791 (19)
S2A—C11A	1.7707 (19)	S1B—S2B	2.0827 (6)
F1A—C8A	1.334 (3)	S2B—C11B	1.7794 (19)
F2A—C8A	1.327 (3)	F1B—C8B	1.346 (3)
F3A—C8A	1.330 (3)	F2B—C8B	1.318 (3)
F4A—C10A	1.320 (3)	F3B—C8B	1.316 (3)
F5A—C10A	1.315 (3)	F4B—C10B	1.325 (3)
F6A—C10A	1.312 (3)	F5B—C10B	1.388 (2)
F4A'—C10A	1.318 (3)	F6B—C10B	1.306 (2)
F5A'—C10A	1.321 (3)	F4B'—C10B	1.336 (3)
F6A'—C10A	1.325 (3)	F5B'—C10B	1.297 (3)
F7A—C18A	1.332 (3)	F6B'—C10B	1.380 (3)
F8A—C18A	1.330 (3)	F7B—C18B	1.342 (3)
F9A—C18A	1.334 (3)	F8B—C18B	1.328 (3)
F10A—C20A	1.326 (3)	F9B—C18B	1.333 (3)
F11A—C20A	1.309 (3)	F10B—C20B	1.329 (2)
F12A—C20A	1.396 (3)	F11B—C20B	1.332 (3)
F10C—C20A	1.315 (3)	F12B—C20B	1.328 (3)
F11C—C20A	1.390 (3)	O1B—C7B	1.207 (3)
F12C—C20A	1.307 (3)	O2B—C9B	1.204 (3)
O1A—C7A	1.213 (3)	O3B—C17B	1.212 (3)
O2A—C9A	1.214 (3)	O4B—C19B	1.212 (2)
O3A—C17A	1.203 (3)	N1B—C7B	1.342 (3)
O4A—C19A	1.210 (3)	N1B—C2B	1.412 (2)
N1A—C7A	1.345 (3)	N1B—H1BN	0.80 (3)
N1A—C2A	1.430 (2)	N2B—C9B	1.353 (3)
N1A—H1AN	0.89 (3)	N2B—C6B	1.410 (3)
N2A—C9A	1.345 (2)	N2B—H2BN	0.84 (3)
N2A—C6A	1.406 (2)	N3B—C17B	1.339 (3)
N2A—H2AN	0.84 (3)	N3B—C12B	1.415 (3)
N3A—C17A	1.349 (3)	N3B—H3BN	0.79 (3)
N3A—C12A	1.418 (2)	N4B—C19B	1.346 (3)
N3A—H3AN	0.84 (3)	N4B—C16B	1.413 (3)
N4A—C19A	1.356 (3)	N4B—H4BN	0.85 (3)
N4A—C16A	1.410 (3)	C1B—C2B	1.404 (3)
N4A—H4AN	0.83 (3)	C1B—C6B	1.410 (3)
C1A—C2A	1.400 (3)	C2B—C3B	1.396 (3)
C1A—C6A	1.409 (3)	C3B—C4B	1.383 (3)
C2A—C3A	1.390 (3)	C3B—H3B	0.9500
C3A—C4A	1.387 (3)	C4B—C5B	1.390 (3)
C3A—H3A	0.9500	C4B—H4B	0.9500
C4A—C5A	1.388 (3)	C5B—C6B	1.388 (3)
C4A—H4A	0.9500	C5B—H5B	0.9500
C5A—C6A	1.399 (3)	C7B—C8B	1.540 (3)
C5A—H5A	0.9500	C9B—C10B	1.517 (3)
C7A—C8A	1.542 (3)	C11B—C12B	1.405 (3)
C9A—C10A	1.536 (3)	C11B—C16B	1.406 (3)
C11A—C12A	1.406 (3)	C12B—C13B	1.396 (3)
C11A—C16A	1.410 (3)	C13B—C14B	1.383 (3)
C12A—C13A	1.395 (3)	C13B—H13B	0.9500
C13A—C14A	1.387 (3)	C14B—C15B	1.386 (3)
C13A—H13A	0.9500	C14B—H14B	0.9500
C14A—C15A	1.386 (3)	C15B—C16B	1.396 (3)
C14A—H14A	0.9500	C15B—H15B	0.9500
C15A—C16A	1.395 (3)	C17B—C18B	1.543 (3)
C15A—H15A	0.9500	C19B—C20B	1.542 (3)
C17A—C18A	1.552 (3)		
			
C1A—S1A—S2A	100.33 (6)	F10A—C20A—C19A	112.6 (3)
C11A—S2A—S1A	99.91 (6)	F11C—C20A—C19A	104.0 (2)
C7A—N1A—C2A	120.29 (17)	F12A—C20A—C19A	108.5 (2)
C7A—N1A—H1AN	122.0 (17)	C1B—S1B—S2B	102.74 (6)
C2A—N1A—H1AN	116.9 (17)	C11B—S2B—S1B	102.73 (6)
C9A—N2A—C6A	129.10 (18)	C7B—N1B—C2B	126.31 (18)
C9A—N2A—H2AN	117.4 (18)	C7B—N1B—H1BN	115.0 (19)
C6A—N2A—H2AN	113.5 (18)	C2B—N1B—H1BN	118.6 (19)
C17A—N3A—C12A	126.14 (17)	C9B—N2B—C6B	127.43 (18)
C17A—N3A—H3AN	116.5 (18)	C9B—N2B—H2BN	116.1 (19)
C12A—N3A—H3AN	117.3 (18)	C6B—N2B—H2BN	116.4 (19)
C19A—N4A—C16A	127.4 (2)	C17B—N3B—C12B	126.10 (18)
C19A—N4A—H4AN	115 (2)	C17B—N3B—H3BN	119 (2)
C16A—N4A—H4AN	117 (2)	C12B—N3B—H3BN	114 (2)
C2A—C1A—C6A	118.86 (17)	C19B—N4B—C16B	127.87 (17)
C2A—C1A—S1A	120.89 (15)	C19B—N4B—H4BN	115.3 (18)
C6A—C1A—S1A	120.25 (14)	C16B—N4B—H4BN	116.4 (18)
C3A—C2A—C1A	120.71 (18)	C2B—C1B—C6B	118.91 (17)
C3A—C2A—N1A	119.54 (18)	C2B—C1B—S1B	119.82 (14)
C1A—C2A—N1A	119.74 (17)	C6B—C1B—S1B	121.27 (15)
C4A—C3A—C2A	119.44 (19)	C3B—C2B—C1B	120.75 (18)
C4A—C3A—H3A	120.3	C3B—C2B—N1B	121.12 (18)
C2A—C3A—H3A	120.3	C1B—C2B—N1B	118.12 (17)
C3A—C4A—C5A	121.44 (19)	C4B—C3B—C2B	118.84 (19)
C3A—C4A—H4A	119.3	C4B—C3B—H3B	120.6
C5A—C4A—H4A	119.3	C2B—C3B—H3B	120.6
C4A—C5A—C6A	119.01 (19)	C3B—C4B—C5B	121.86 (19)
C4A—C5A—H5A	120.5	C3B—C4B—H4B	119.1
C6A—C5A—H5A	120.5	C5B—C4B—H4B	119.1
C5A—C6A—N2A	122.99 (18)	C6B—C5B—C4B	119.32 (19)
C5A—C6A—C1A	120.50 (18)	C6B—C5B—H5B	120.3
N2A—C6A—C1A	116.43 (17)	C4B—C5B—H5B	120.3
O1A—C7A—N1A	125.97 (19)	C5B—C6B—N2B	122.50 (18)
O1A—C7A—C8A	117.83 (19)	C5B—C6B—C1B	120.33 (18)
N1A—C7A—C8A	116.09 (18)	N2B—C6B—C1B	117.12 (17)
F2A—C8A—F3A	108.47 (17)	O1B—C7B—N1B	127.53 (19)
F2A—C8A—F1A	107.90 (18)	O1B—C7B—C8B	118.70 (18)
F3A—C8A—F1A	107.61 (19)	N1B—C7B—C8B	113.76 (18)
F2A—C8A—C7A	110.34 (19)	F3B—C8B—F2B	108.7 (2)
F3A—C8A—C7A	113.38 (17)	F3B—C8B—F1B	106.3 (2)
F1A—C8A—C7A	108.97 (17)	F2B—C8B—F1B	107.9 (2)
O2A—C9A—N2A	128.4 (2)	F3B—C8B—C7B	110.79 (19)
O2A—C9A—C10A	119.01 (17)	F2B—C8B—C7B	110.9 (2)
N2A—C9A—C10A	112.60 (17)	F1B—C8B—C7B	112.12 (18)
F6A—C10A—F5A	108.6 (3)	O2B—C9B—N2B	128.4 (2)
F6A—C10A—F4A	109.3 (3)	O2B—C9B—C10B	118.72 (18)
F5A—C10A—F4A	107.0 (3)	N2B—C9B—C10B	112.80 (17)
F4A'—C10A—F5A'	107.5 (3)	F6B—C10B—F4B	110.8 (3)
F4A'—C10A—F6A'	107.3 (3)	F5B'—C10B—F4B'	110.9 (4)
F5A'—C10A—F6A'	105.5 (3)	F5B'—C10B—F6B'	105.2 (3)
F6A—C10A—C9A	109.6 (3)	F4B'—C10B—F6B'	102.2 (4)
F5A—C10A—C9A	110.5 (3)	F6B—C10B—F5B	103.9 (2)
F4A'—C10A—C9A	114.1 (3)	F4B—C10B—F5B	105.7 (3)
F4A—C10A—C9A	111.7 (2)	F5B'—C10B—C9B	118.9 (3)
F5A'—C10A—C9A	111.1 (3)	F6B—C10B—C9B	114.0 (2)
F6A'—C10A—C9A	110.9 (3)	F4B—C10B—C9B	116.2 (2)
C12A—C11A—C16A	119.68 (17)	F4B'—C10B—C9B	113.5 (3)
C12A—C11A—S2A	120.17 (15)	F6B'—C10B—C9B	104.1 (3)
C16A—C11A—S2A	120.13 (15)	F5B—C10B—C9B	104.97 (19)
C13A—C12A—C11A	119.99 (18)	C12B—C11B—C16B	119.05 (17)
C13A—C12A—N3A	121.37 (18)	C12B—C11B—S2B	119.72 (15)
C11A—C12A—N3A	118.64 (17)	C16B—C11B—S2B	121.21 (15)
C14A—C13A—C12A	119.02 (19)	C13B—C12B—C11B	120.62 (19)
C14A—C13A—H13A	120.5	C13B—C12B—N3B	121.13 (18)
C12A—C13A—H13A	120.5	C11B—C12B—N3B	118.17 (17)
C15A—C14A—C13A	122.35 (19)	C14B—C13B—C12B	118.80 (19)
C15A—C14A—H14A	118.8	C14B—C13B—H13B	120.6
C13A—C14A—H14A	118.8	C12B—C13B—H13B	120.6
C14A—C15A—C16A	118.84 (19)	C13B—C14B—C15B	122.18 (19)
C14A—C15A—H15A	120.6	C13B—C14B—H14B	118.9
C16A—C15A—H15A	120.6	C15B—C14B—H14B	118.9
C15A—C16A—N4A	122.16 (18)	C14B—C15B—C16B	118.92 (19)
C15A—C16A—C11A	120.08 (19)	C14B—C15B—H15B	120.5
N4A—C16A—C11A	117.76 (18)	C16B—C15B—H15B	120.5
O3A—C17A—N3A	128.1 (2)	C15B—C16B—C11B	120.40 (18)
O3A—C17A—C18A	118.51 (19)	C15B—C16B—N4B	122.04 (18)
N3A—C17A—C18A	113.40 (18)	C11B—C16B—N4B	117.54 (17)
F8A—C18A—F7A	108.03 (19)	O3B—C17B—N3B	128.0 (2)
F8A—C18A—F9A	106.99 (19)	O3B—C17B—C18B	119.23 (19)
F7A—C18A—F9A	107.46 (18)	N3B—C17B—C18B	112.78 (18)
F8A—C18A—C17A	111.23 (18)	F8B—C18B—F9B	108.00 (17)
F7A—C18A—C17A	110.01 (18)	F8B—C18B—F7B	107.65 (18)
F9A—C18A—C17A	112.91 (17)	F9B—C18B—F7B	107.53 (18)
O4A—C19A—N4A	128.3 (2)	F8B—C18B—C17B	109.90 (18)
O4A—C19A—C20A	119.7 (2)	F9B—C18B—C17B	110.90 (18)
N4A—C19A—C20A	111.95 (19)	F7B—C18B—C17B	112.69 (17)
F12C—C20A—F10C	114.0 (4)	O4B—C19B—N4B	128.55 (19)
F11A—C20A—F10A	110.9 (3)	O4B—C19B—C20B	117.57 (18)
F12C—C20A—F11C	102.2 (3)	N4B—C19B—C20B	113.82 (17)
F10C—C20A—F11C	103.8 (3)	F12B—C20B—F10B	108.42 (18)
F11A—C20A—F12A	101.7 (3)	F12B—C20B—F11B	107.86 (17)
F10A—C20A—F12A	102.1 (3)	F10B—C20B—F11B	107.97 (19)
F12C—C20A—C19A	116.7 (3)	F12B—C20B—C19B	108.87 (18)
F11A—C20A—C19A	119.0 (3)	F10B—C20B—C19B	113.46 (17)
F10C—C20A—C19A	113.8 (3)	F11B—C20B—C19B	110.10 (17)
			
S2A—S1A—C1A—C2A	102.74 (15)	N4A—C19A—C20A—F11C	−94.1 (3)
S2A—S1A—C1A—C6A	−76.26 (15)	O4A—C19A—C20A—F12A	−123.9 (3)
C6A—C1A—C2A—C3A	1.5 (3)	N4A—C19A—C20A—F12A	57.9 (3)
S1A—C1A—C2A—C3A	−177.49 (16)	S2B—S1B—C1B—C2B	104.09 (15)
C6A—C1A—C2A—N1A	−179.48 (17)	S2B—S1B—C1B—C6B	−76.37 (16)
S1A—C1A—C2A—N1A	1.5 (3)	C6B—C1B—C2B—C3B	−0.1 (3)
C7A—N1A—C2A—C3A	−119.4 (2)	S1B—C1B—C2B—C3B	179.48 (15)
C7A—N1A—C2A—C1A	61.6 (3)	C6B—C1B—C2B—N1B	−178.92 (17)
C1A—C2A—C3A—C4A	−0.2 (3)	S1B—C1B—C2B—N1B	0.6 (2)
N1A—C2A—C3A—C4A	−179.21 (19)	C7B—N1B—C2B—C3B	31.0 (3)
C2A—C3A—C4A—C5A	−1.0 (3)	C7B—N1B—C2B—C1B	−150.1 (2)
C3A—C4A—C5A—C6A	0.9 (3)	C1B—C2B—C3B—C4B	0.3 (3)
C4A—C5A—C6A—N2A	177.10 (19)	N1B—C2B—C3B—C4B	179.12 (19)
C4A—C5A—C6A—C1A	0.5 (3)	C2B—C3B—C4B—C5B	−0.1 (3)
C9A—N2A—C6A—C5A	−1.1 (3)	C3B—C4B—C5B—C6B	−0.3 (3)
C9A—N2A—C6A—C1A	175.62 (19)	C4B—C5B—C6B—N2B	177.99 (19)
C2A—C1A—C6A—C5A	−1.7 (3)	C4B—C5B—C6B—C1B	0.6 (3)
S1A—C1A—C6A—C5A	177.34 (15)	C9B—N2B—C6B—C5B	−6.8 (3)
C2A—C1A—C6A—N2A	−178.48 (17)	C9B—N2B—C6B—C1B	170.72 (19)
S1A—C1A—C6A—N2A	0.5 (2)	C2B—C1B—C6B—C5B	−0.4 (3)
C2A—N1A—C7A—O1A	3.0 (3)	S1B—C1B—C6B—C5B	−179.92 (15)
C2A—N1A—C7A—C8A	−172.99 (17)	C2B—C1B—C6B—N2B	−177.92 (17)
O1A—C7A—C8A—F2A	54.3 (3)	S1B—C1B—C6B—N2B	2.5 (2)
N1A—C7A—C8A—F2A	−129.3 (2)	C2B—N1B—C7B—O1B	−1.1 (4)
O1A—C7A—C8A—F3A	176.16 (19)	C2B—N1B—C7B—C8B	179.78 (19)
N1A—C7A—C8A—F3A	−7.5 (3)	O1B—C7B—C8B—F3B	26.6 (3)
O1A—C7A—C8A—F1A	−64.0 (3)	N1B—C7B—C8B—F3B	−154.2 (2)
N1A—C7A—C8A—F1A	112.4 (2)	O1B—C7B—C8B—F2B	−94.2 (3)
C6A—N2A—C9A—O2A	6.0 (4)	N1B—C7B—C8B—F2B	85.0 (2)
C6A—N2A—C9A—C10A	−173.50 (19)	O1B—C7B—C8B—F1B	145.1 (2)
O2A—C9A—C10A—F6A	−77.4 (4)	N1B—C7B—C8B—F1B	−35.7 (3)
N2A—C9A—C10A—F6A	102.1 (4)	C6B—N2B—C9B—O2B	6.5 (4)
O2A—C9A—C10A—F5A	42.3 (5)	C6B—N2B—C9B—C10B	−170.46 (18)
N2A—C9A—C10A—F5A	−138.2 (4)	O2B—C9B—C10B—F5B'	−45.7 (5)
O2A—C9A—C10A—F4A'	−169.2 (5)	N2B—C9B—C10B—F5B'	131.6 (5)
N2A—C9A—C10A—F4A'	10.3 (5)	O2B—C9B—C10B—F6B	33.6 (3)
O2A—C9A—C10A—F4A	161.2 (3)	N2B—C9B—C10B—F6B	−149.1 (3)
N2A—C9A—C10A—F4A	−19.2 (4)	O2B—C9B—C10B—F4B	164.3 (3)
O2A—C9A—C10A—F5A'	−47.4 (4)	N2B—C9B—C10B—F4B	−18.5 (4)
N2A—C9A—C10A—F5A'	132.1 (4)	O2B—C9B—C10B—F4B'	−178.9 (4)
O2A—C9A—C10A—F6A'	69.5 (4)	N2B—C9B—C10B—F4B'	−1.6 (5)
N2A—C9A—C10A—F6A'	−110.9 (4)	O2B—C9B—C10B—F6B'	70.8 (4)
S1A—S2A—C11A—C12A	103.05 (15)	N2B—C9B—C10B—F6B'	−111.9 (4)
S1A—S2A—C11A—C16A	−75.07 (16)	O2B—C9B—C10B—F5B	−79.4 (3)
C16A—C11A—C12A—C13A	−0.7 (3)	N2B—C9B—C10B—F5B	97.9 (3)
S2A—C11A—C12A—C13A	−178.81 (15)	S1B—S2B—C11B—C12B	97.17 (15)
C16A—C11A—C12A—N3A	179.69 (17)	S1B—S2B—C11B—C16B	−84.67 (16)
S2A—C11A—C12A—N3A	1.6 (2)	C16B—C11B—C12B—C13B	1.9 (3)
C17A—N3A—C12A—C13A	27.8 (3)	S2B—C11B—C12B—C13B	−179.90 (16)
C17A—N3A—C12A—C11A	−152.5 (2)	C16B—C11B—C12B—N3B	−174.94 (17)
C11A—C12A—C13A—C14A	2.0 (3)	S2B—C11B—C12B—N3B	3.3 (2)
N3A—C12A—C13A—C14A	−178.39 (19)	C17B—N3B—C12B—C13B	32.1 (3)
C12A—C13A—C14A—C15A	−1.6 (3)	C17B—N3B—C12B—C11B	−151.1 (2)
C13A—C14A—C15A—C16A	−0.2 (3)	C11B—C12B—C13B—C14B	−1.8 (3)
C14A—C15A—C16A—N4A	−179.12 (19)	N3B—C12B—C13B—C14B	174.96 (19)
C14A—C15A—C16A—C11A	1.5 (3)	C12B—C13B—C14B—C15B	0.4 (3)
C19A—N4A—C16A—C15A	−2.5 (3)	C13B—C14B—C15B—C16B	0.8 (3)
C19A—N4A—C16A—C11A	176.9 (2)	C14B—C15B—C16B—C11B	−0.7 (3)
C12A—C11A—C16A—C15A	−1.1 (3)	C14B—C15B—C16B—N4B	177.97 (19)
S2A—C11A—C16A—C15A	177.03 (16)	C12B—C11B—C16B—C15B	−0.7 (3)
C12A—C11A—C16A—N4A	179.51 (18)	S2B—C11B—C16B—C15B	−178.83 (15)
S2A—C11A—C16A—N4A	−2.4 (3)	C12B—C11B—C16B—N4B	−179.36 (17)
C12A—N3A—C17A—O3A	−3.4 (4)	S2B—C11B—C16B—N4B	2.5 (2)
C12A—N3A—C17A—C18A	177.69 (18)	C19B—N4B—C16B—C15B	−2.0 (3)
O3A—C17A—C18A—F8A	−87.1 (3)	C19B—N4B—C16B—C11B	176.69 (19)
N3A—C17A—C18A—F8A	91.9 (2)	C12B—N3B—C17B—O3B	5.0 (4)
O3A—C17A—C18A—F7A	32.5 (3)	C12B—N3B—C17B—C18B	−173.19 (18)
N3A—C17A—C18A—F7A	−148.47 (19)	O3B—C17B—C18B—F8B	−82.9 (3)
O3A—C17A—C18A—F9A	152.6 (2)	N3B—C17B—C18B—F8B	95.5 (2)
N3A—C17A—C18A—F9A	−28.4 (3)	O3B—C17B—C18B—F9B	36.4 (3)
C16A—N4A—C19A—O4A	−1.1 (4)	N3B—C17B—C18B—F9B	−145.18 (18)
C16A—N4A—C19A—C20A	176.9 (2)	O3B—C17B—C18B—F7B	157.0 (2)
O4A—C19A—C20A—F12C	−164.1 (4)	N3B—C17B—C18B—F7B	−24.6 (3)
N4A—C19A—C20A—F12C	17.7 (5)	C16B—N4B—C19B—O4B	4.9 (4)
O4A—C19A—C20A—F11A	120.7 (5)	C16B—N4B—C19B—C20B	−172.29 (18)
N4A—C19A—C20A—F11A	−57.5 (4)	O4B—C19B—C20B—F12B	−71.4 (2)
O4A—C19A—C20A—F10C	−28.2 (4)	N4B—C19B—C20B—F12B	106.10 (19)
N4A—C19A—C20A—F10C	153.6 (4)	O4B—C19B—C20B—F10B	167.8 (2)
O4A—C19A—C20A—F10A	−11.6 (5)	N4B—C19B—C20B—F10B	−14.7 (3)
N4A—C19A—C20A—F10A	170.2 (4)	O4B—C19B—C20B—F11B	46.7 (3)
O4A—C19A—C20A—F11C	84.1 (3)	N4B—C19B—C20B—F11B	−135.85 (18)

### Hydrogen-bond geometry (Å, º)

**Table:**

*D*—H···*A*	*D*—H	H···*A*	*D*···*A*	*D*—H···*A*
N1*A*—H1*AN*···O4*B*	0.89 (3)	2.19 (3)	2.988 (2)	149 (2)
N2*A*—H2*AN*···S1*A*	0.84 (3)	2.42 (3)	2.9425 (18)	121 (2)
N2*A*—H2*AN*···S2*A*	0.84 (3)	2.96 (3)	3.4543 (18)	120 (2)
N2*A*—H2*AN*···F4*A*	0.84 (3)	2.16 (3)	2.574 (4)	110 (2)
N2*A*—H2*AN*···F4*A*′	0.84 (3)	2.19 (3)	2.600 (5)	110 (2)
N3*A*—H3*AN*···O1*A*^i^	0.84 (3)	2.12 (3)	2.857 (2)	146 (2)
N4*A*—H4*AN*···S2*A*	0.83 (3)	2.52 (3)	2.9697 (19)	116 (2)
N4*A*—H4*AN*···F12*C*	0.83 (3)	2.21 (3)	2.643 (4)	113 (2)
C5*A*—H5*A*···O2*A*	0.95	2.34	2.952 (3)	122
C13*A*—H13*A*···F5*B*′^ii^	0.95	2.54	3.207 (4)	127
C15*A*—H15*A*···O4*A*	0.95	2.25	2.883 (3)	123
N1*B*—H1*BN*···O2*A*^iii^	0.80 (3)	2.42 (3)	2.983 (2)	128 (2)
N2*B*—H2*BN*···S1*B*	0.84 (3)	2.51 (3)	2.9892 (18)	117 (2)
N2*B*—H2*BN*···F4*B*	0.84 (3)	2.23 (3)	2.657 (4)	112 (2)
N2*B*—H2*BN*···F4*B*′	0.84 (3)	2.13 (3)	2.574 (7)	113 (2)
N3*B*—H3*BN*···O1*B*^iv^	0.79 (3)	2.24 (3)	2.848 (2)	135 (3)
N4*B*—H4*BN*···S2*B*	0.85 (3)	2.53 (3)	2.9936 (17)	116 (2)
N4*B*—H4*BN*···F10*B*	0.85 (3)	2.20 (3)	2.634 (2)	112 (2)
N4*B*—H4*BN*···O3*B*^iv^	0.85 (3)	2.47 (3)	3.032 (2)	125 (2)
C5*B*—H5*B*···O2*B*	0.95	2.27	2.896 (3)	122
C15*B*—H15*B*···O3*A*	0.95	2.56	3.229 (3)	128
C15*B*—H15*B*···O4*B*	0.95	2.27	2.898 (3)	123

Symmetry codes: (i) −*x*+1/2, *y*−1/2, −*z*+1/2; (ii) −*x*+3/2, *y*−1/2, −*z*+1/2; (iii) *x*+1/2, −*y*+3/2, *z*−1/2; (iv) −*x*+3/2, *y*+1/2, −*z*+1/2.
