# Supplementary figures and images for: Crystal structure of N-(2-{[2,6-bis­(2,2,2-tri­fluoro­acetamido)­phen­yl]disulfan­yl}-3-(2,2,2-tri­fluoro­acetamido)­phen­yl)-2,2,2-tri­fluoro­acetamide
Source: Acta Crystallogr E Crystallogr Commun. 2015 Aug 6;71(Pt 9):o639–40. doi: 10.1107/S2056989015014231 (PMC4555375; doi:10.1107/S2056989015014231)

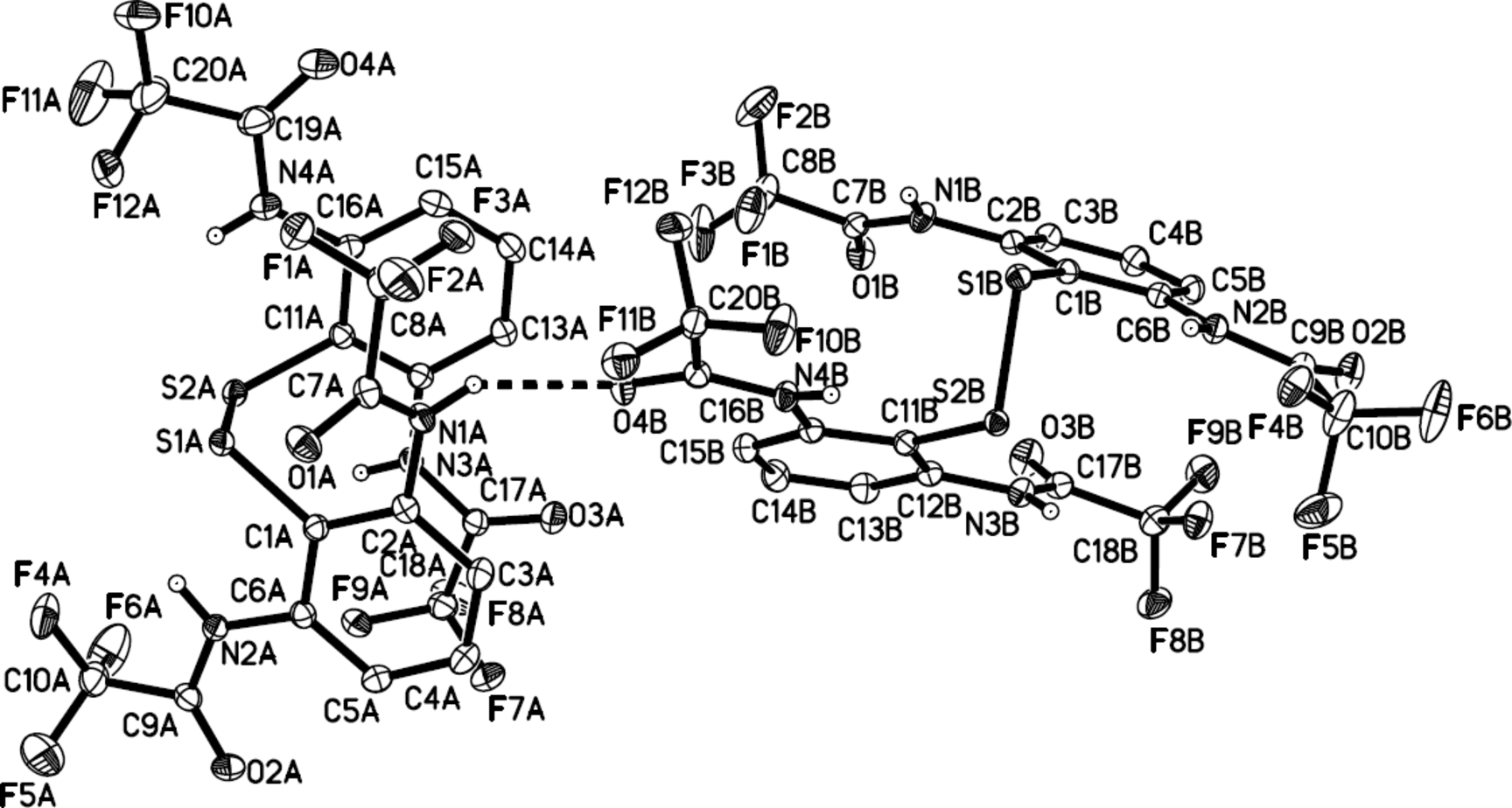

Supplement: Supplementary file 4 [file e-71-0o639-fig1.tif]

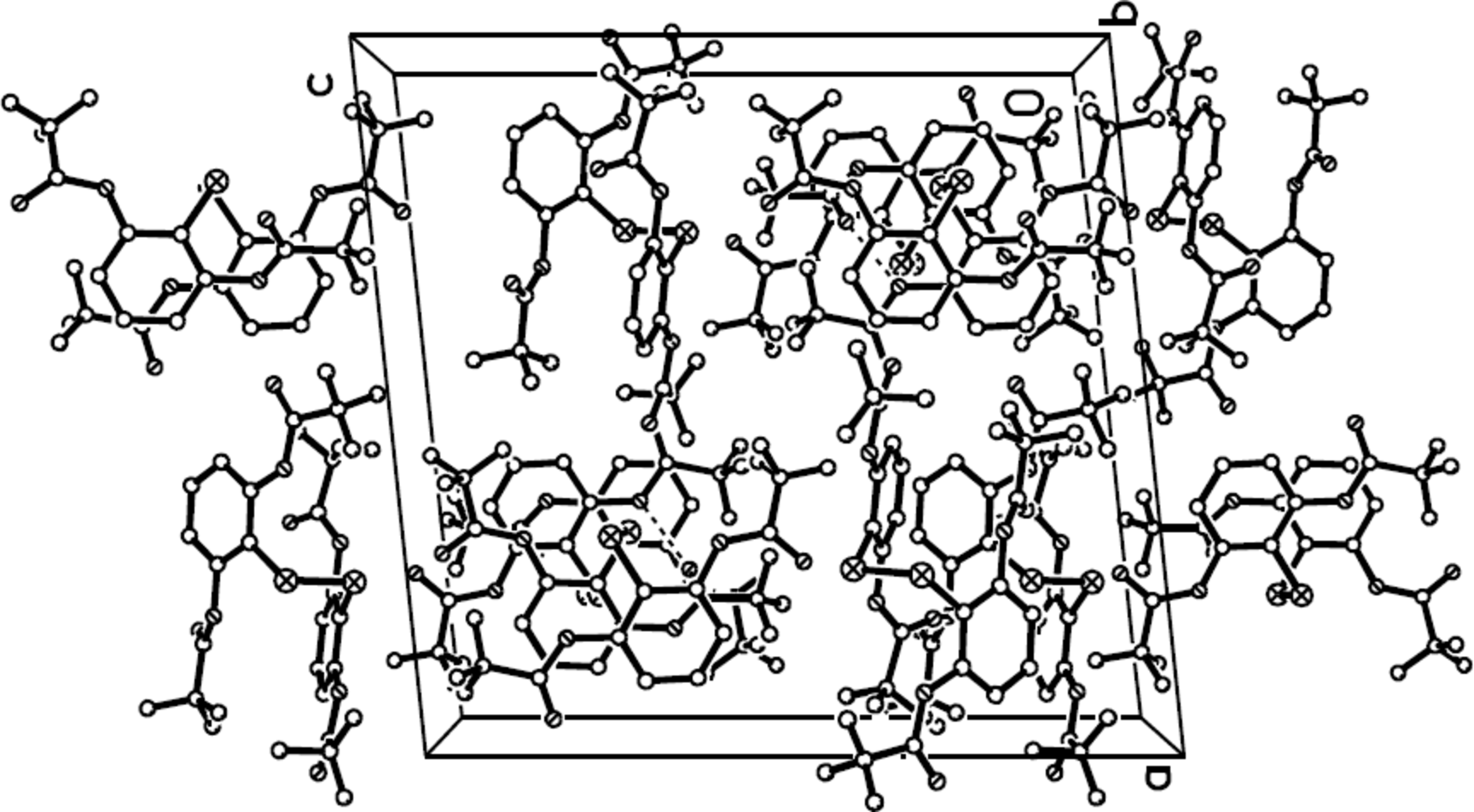

Supplement: Supplementary file 5 [file e-71-0o639-fig2.tif]
